# Function of T_reg_ Cells Decreased in Patients With Systemic Lupus Erythematosus Due To the Effect of Prolactin

**DOI:** 10.1097/MD.0000000000002384

**Published:** 2016-02-08

**Authors:** María Victoria Legorreta-Haquet, Karina Chávez-Rueda, Luis Chávez-Sánchez, Hernando Cervera-Castillo, Edgar Zenteno-Galindo, Leonor Barile-Fabris, Rubén Burgos-Vargas, Everardo Álvarez-Hernández, Francisco Blanco-Favela

**Affiliations:** From the Unidad de Investigación Médica en Inmunología, Hospital de Pediatría, C.M.N. “Siglo XXI”, IMSS, Mexico (L-HMV, C-RK, C-SL, B-FF); Departamento de Reumatología, Clínica 25, IMSS, Mexico (C-CH); Departamento de Bioquímica, Universidad Nacional Autónoma de México, Mexico (Z-GE); Departamento de Reumatología, Hospital de Especialidades, Centro Médico Nacional “Siglo XXI”, Mexico (B-FL); Servicio de Reumatología, Hospital General de México, “Dr. Eduardo Liceaga”, Mexico (B-VR, Á-HE).

## Abstract

Prolactin has different functions, including cytokine secretion and inhibition of the suppressor effect of regulatory T (T_reg_) cells in healthy individuals. Systemic lupus erythematosus (SLE) is an autoimmune disease characterized by defects in the functions of B, T, and T_reg_ cells. Prolactin plays an important role in the physiopathology of SLE. Our objective was to establish the participation of prolactin in the regulation of the immune response mediated by T_reg_ cells from patients with SLE. CD4^+^CD25^hi^CD127^−/low^ cells were purified using magnetic beads and the relative expression of prolactin receptor was measured. The functional activity was evaluated by proliferation assay and cytokine secretion in activated cells, in the presence and absence of prolactin. We found that both percentage and function of T_reg_ cells decrease in SLE patients compared to healthy individuals with statistical significance. The prolactin receptor is constitutively expressed on T_reg_ and effector T (T_eff_) cells in SLE patients, and this expression is higher than in healthy individuals. The expression of this receptor differs in inactive and active patients: in the former, the expression is higher in T_reg_ cells than in T_eff_ cells, similar to healthy individuals, whereas there is no difference in the expression between T_reg_ and T_eff_ cells from active patients. In T_reg_:T_eff_ cell cocultures, addition of prolactin decreases the suppressor effect exerted by T_reg_ cells and increases IFNγ secretion. Our results suggest that prolactin plays an important role in the activation of the disease in inactive patients by decreasing the suppressor function exerted by T_reg_ cells over T_eff_ cells, thereby favoring an inflammatory microenvironment.

## Abstract 2

Supplemental Digital Content is available in the text

## INTRODUCTION

Systemic lupus erythematosus (SLE) is an autoimmune rheumatic disease characterized by widespread inflammation, alteration in T cell activation, and overproduction of autoantibodies. This disease is most commonly observed in women. The course of the disease is characterized by remissions and exacerbation. The exacerbation of the disease has been linked to the activity of the immune system.^1^ Autoreactive T cells assist autoreactive B cells and infiltrate into the target organs to promote inflammation via cytokine secretion, which causes damage. Thus, autoreactive T cells are key players in the pathogenesis of SLE.^2^

Hyperprolactinemia has been reported in several autoimmune diseases, including SLE.^3–6^ Prolactin (PRL) can be synthesized in an extra-pituitary fashion by cells from the immune system, such as B and T cells, which also express the PRL receptor.^7,8^ During an immune response, PRL promotes the proliferation, growth, activation, and differentiation of T cells^9,10^ and intervenes in the expression of CD69 and CD154 by CD4^+^ T cells.^11^ In human CD4^+^ T cell cultures activated with phorbol myristate acetate and subjected to PRL blockade by using an anti-PRL antibody, IL2 and IFNγ secretion is decreased, indicating a role for PRL in the regulation of cytokine secretion.^12^ Furthermore, PRL can decrease the function of regulatory T (T_reg_) cells^13^ in healthy individuals. These studies show the importance of PRL in the regulation of the immune system.

The pathogenesis of SLE involves complex interactions between genetic and environmental factors and the adaptive and innate immune systems. The breakdown of immunologic self-tolerance results in the development of autoimmune diseases.^14,15^ Other alterations could also be involved in regulating the immune response mediated by T_reg_ cells. There are 2 types of T_reg_ cells: natural T_reg_ cells, which are generated in the thymus, and inducible T_reg_ cells, which are generated in peripheral sites. Both cells exhibit the same CD4^+^CD25^hi^CD127^low/−^FoxP3^+^ phenotype.^16,17^ T_reg_ cells exert an inhibitory effect on CD4^+^CD25^−^CD127^+^ conventional or effector T (T_eff_) cells.^18^ A numerical defect in T_reg_ cells has been observed in autoimmune pathologies such as thyroiditis^19^ and diabetes,^20^ whereas in SLE, decreased^21–26^ as well as normal^27–30^ T_reg_ cell numbers have been reported. Moreover, in SLE patients, conventional T cells exhibit reduced sensitivity to T_reg_ cell inhibition.^22,31,32^

The objective of our work was to determine whether PRL participates in the regulation of the immune response mediated by T_reg_ cells in patients with SLE. We found that both percentage and function of T_reg_ (CD4^+^CD25^hi^CD127^−/low^FoxP3^+^) cells were decreased in SLE patients compared to healthy individuals. The expression of PRL receptor was found to be constitutive in both T_reg_ and T_eff_ cells in patients with SLE and this expression was increased compared to that in healthy individuals. PRL receptor expression varied among SLE patients; in inactive patients, the expression of the receptor was higher in T_reg_ cells compared to T_eff_ cells, similar to what was observed in healthy individuals. However, there was no difference in the expression of the receptor between T_reg_ and T_eff_ cells among active SLE patients. We also found that PRL affects the function of T_reg_ cells. The addition of prolactin to T_reg_:T_eff_ cocultures decreased the suppressor effect in T_reg_ cells and increased IFNγ secretion. These results suggest that PRL increases IFNγ secretion, favoring an inflammatory environment, and decreases the suppressor function of T_reg_ cells; this, in addition to the decrease in the number of T_reg_ cells, contributes to the expansion of autoreactive lymphocytes, favoring disease activation.

## METHODS

### Study Group

The Ethics Committee of Human Research of the Instituto Mexicano del Seguro Social (IMSS) and the Ethics and Research Committees of the Hospital General de México approved this study (2009-785-028). It was conducted according to the Declaration of Helsinki. Informed consent was obtained from all participants. The samples were obtained from 17 healthy women in the reproductive age (18–50 years) without menstrual disorders and with normal levels of serum prolactin (<20 ng/ml). Since T_reg_ is a rare cell population, the cells from 1 patient are inadequate for all experiments; therefore, from a total of 68 patients with SLE (25–50 years of age), we used samples from an average of 13 patients with inactive lupus and 13 patients with active lupus for each experiment. All patients with SLE fulfilled the American College of Rheumatology (ACR) criteria for SLE.^33^

Disease activity was measured by SLEDAI (systemic lupus erythematosus disease activity index). Inactive lupus was considered when the SLEDAI value was equal to 0; lupus was considered to be active when the SLEDAI value was ≥4. The samples were obtained between 08:00 and 11:00 am from the cubital vein.

### Prolactin

The human PRL used in this study was kindly provided by Dr. A.F. Parlow, from the National Hormone & Pituitary Program (NHPP; Harbor UCLA Medical Center, Los Angeles, CA).

### Antibodies

The following antibodies were used: mouse anti-human CD4-APC (OKT4), CD25-PE-Cy5 (BC96), CD127-FITC (eBioRDR5), FoxP3-PE (PCH101), and CD25-APC (BC96), all from eBioscience (San Diego, CA); mouse anti-PRL receptor (ECD, 1A2B1) from Invitrogen (Carlsbad, CA); and Biotin Rat Anti-Mouse IgG2b (R12-3) from BD Pharmingen (San Jose, CA). The biotinylated secondary antibody was detected using streptavidin–phycoerythrin–Cy5.5 from BD Biosciences (Mountain View, CA).

### T_reg_ and T_eff_ Cell Purification

Peripheral blood mononuclear cells (PBMCs) were isolated from whole blood samples by density centrifugation using Lymphoprep (Axis Shield, Oslo, Norway). T_reg_ cells were isolated from PBMCs by using a CD4^+^CD25^+^CD127^dim/−^ Regulatory T cell Isolation Kit II (Miltenyi Biotec, Bergish Gladbach, Germany), according to the manufacturer's instructions. The purity of the cells ranged from 93% to 97% (Supplemental Content 1).

### Cell Culture and Proliferation Assays

Cells were cultured in AIM-V liquid medium (Gibco BRL, NY, New York) supplemented with 50 units/ml penicillin and 50 μg/ml streptomycin (Gibco BRL). T_reg_ cells (CD4^+^CD25^hi^CD127^low/−^) were plated at a density of 4.0 × 10^4^ cells/well in 96-well U-bottomed plates (Nunc, Roskilde, Denmark) with or without 8.0 × 10^4^ T_eff_ cells (CD4^+^CD25^−^CD127^+^) and cultured in synthetic serum-free medium (AIM-V, Gibco BRL). We standardized the optimum ratio of T_reg_:T_eff_ cells required to generate a response by using a standard curve illustrating the following ratios: 0.5:1, 1:1, 2:1, and 4:1. The suppressor effect was observed under all conditions; thus, we decided to use a 0.5:1 T_reg_:T_eff_ cell ratio, on the basis of the percentage of circulating T_reg_ cells and the feasibility of obtaining sufficient quantities for all tests.

T_reg_ Suppression Inspector human (anti-CD2/CD3/CD28 beads; Miltenyi Biotec, Germany) was used for the functional characterization of human T_reg_ cells by in vitro suppression assays in the presence and absence of 50 ng/ml human PRL (NHPP, Los Angeles, CA). The concentrations of Inspector and PRL were obtained using a dose–response curve. Cells were cultured for 5 days, and 1 μCi [^3^H]-thymidine (Hartmann Analytical, Braunschweig, Germany) was added 18 hours before harvesting. Thymidine incorporation was determined using a liquid scintillation analyzer (Packard 1900 TR, Meriden, Connecticut), and the percentage of proliferation suppression was determined. All conditions were previously standardized and optimized.

### Cytokine Detection

Cell culture supernatants were collected on day 5, and cytokine levels were measured using a commercial BD Cytometric Bead Array (CBA) Human Th1/Th2/Th17 Cytokine Kit (IL2, IL4, IL10, IL6, TNF, IFNγ, and IL17A) by BD Biosciences.

### Real-Time PCR Assay

Total RNA was extracted from purified T_reg_ and T_eff_ cells by using TRIzol Reagent (Invitrogen), according to the manufacturer's instructions. RNA concentration was determined using UV spectrophotometry, and 1 μg of total RNA was used to generate cDNA with SuperScript II reverse transcriptase (Invitrogen). The PRL receptor and β actin were then amplified by real-time PCR using a LightCycler TaqMan Master kit (Roche Diagnostic, Mannheim, Germany), hydrolysis probes, and primers designed by Roche Diagnostic; all reactions were performed according to the manufacturer's specifications. The primers and probes used are as follows: number 8 probe from the Universal Probe Library for PRL receptor determination, forward primer CTT TCC ACA TGA ACC CTG AAG and reverse primer GCA GAT GCC ACA TTT TCC TT, and number 64 probe from Universal Probe Library for β-actin determination, forward primer CCA ACC GCG AGA AGA TGA and reverse primer CCA GAG GCG TAC AGG GAT AG. Reactions were carried out in a final volume of 10 μl, and a LightCycler 1.5 instrument was used (Roche Diagnostic). The PCR conditions were as follows: 10 minutes at 95°C, followed by 45 cycles of 10 seconds at 95°C, 30 seconds at 59°C, and 1 seconds at 72°C, with a final cycle for 30 seconds at 40°C. The samples were normalized to β-actin gene expression. The relative expression of PRL and its receptor was calculated using the 2^ΔCT^ formula.

### Cell Surface Staining and Flow Cytometry

To determine the percentage of peripheral blood T_reg_ cells, PBMCs were incubated with fluorescently labeled antibodies (anti-CD4, CD25, CD127, and PRL receptor or unrelated antibody) for 20 minutes at 4°C in staining buffer (phosphate-buffered saline [PBS] with 0.5% bovine serum albumin [BSA] and 0.01% sodium azide). The cells were then washed and fixed in 2% PBS–paraformaldehyde (Sigma Aldrich, St. Louis, MO). Data were obtained using a MACSQuant Analyzer 10 flow cytometer (Miltenyi Biotec, Auburn, CA) and analyzed with FlowJo software (Tree Star, Ashland, OR).

### Intracellular Staining for FoxP3

After the superficial staining, the cells were fixed and permeabilized with the Foxp3 Staining Buffer Set (eBioscience) for 18 hours, and stained with fluorescent antibodies. After washing, the stained cells were assayed in a MACSQuant Analyzer 10 flow cytometer and the data were analyzed with FlowJo software.

### Statistical Analysis

Statistical analysis was performed using the SPSS package, version 20.0 (SPSS, Inc., Chicago, IL). Normality of the data was checked using the Kolmogorov–Smirnoff test, followed by an analysis using the relevant parametric or nonparametric test. The suppressor function among the groups was assessed using the Kruskal–Wallis test. Comparisons between individual groups were tested using the unpaired Mann–Whitney *U* or paired Wilcoxon matched-pairs test, at a significance level of *P* < 0.05.

## RESULTS

### Percentage of T_reg_ Cells

**FIGURE 1:**
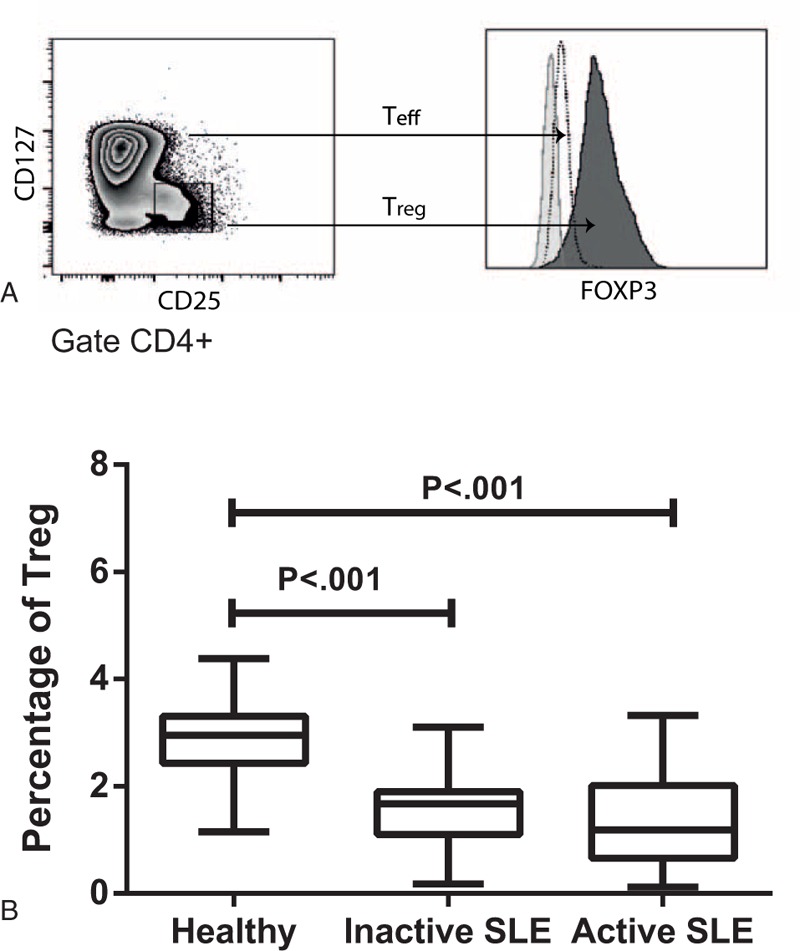
Percentage of T_reg_ CD4^+^CD25^hi^CD127^−/low^FOXP3^+^ cells, PBMCs were stained with CD4, CD25, CD127, and FOXP3 antibodies, and the percentage of T_reg_ cells was determined by flow cytometry. (A) Analysis strategy for determining the percentage of T_reg_ cells from the CD4^+^ gate. (B) Percentage of T_reg_ cells in healthy individuals as well as patients with active and inactive SLE. The graph shows the median value; *P* < 0.001.

The percentage of T_reg_ (CD4^+^CD25^hi^CD127^low/−^FoxP3^+^) cells was determined based on PBMCs from healthy individuals and SLE patients (active and inactive). We found that the percentage of T_reg_ cells decreased in a statistically significant way (*P* < 0.001) in patients with active and inactive SLE, compared to healthy individuals (χ¯=2.95%), but no difference was observed between the inactive (χ¯=1.67%) and active (χ¯=1.19%) patients, suggesting that the number of T_reg_ cells is less in SLE patients (active and inactive) (Figure 1).

### PRL Receptor Expression in T_reg_ and T_eff_ Cells

**FIGURE 2:**
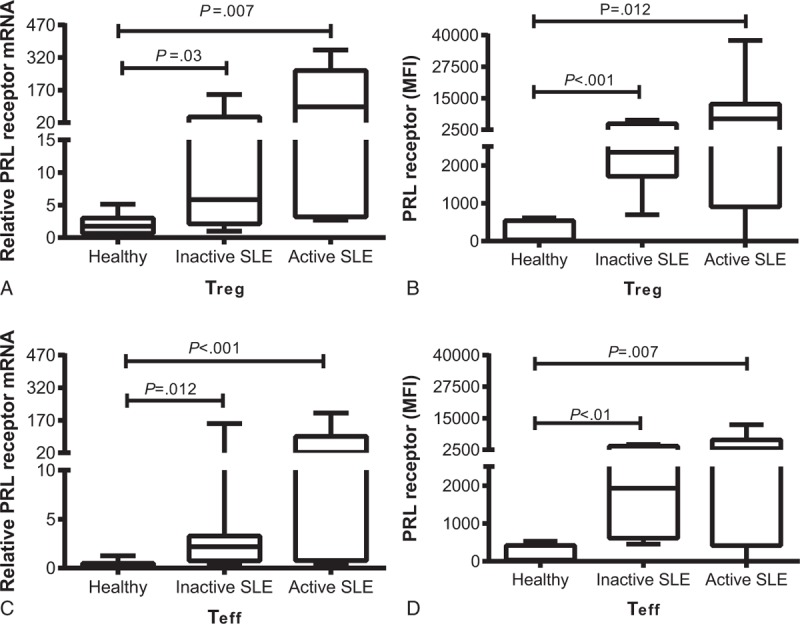
Expression of PRL receptor, T_eff_ (CD4^+^CD25^−^CD127^+^) and T_reg_ (CD4^+^CD25^hi^CD127^−/low^) cell subpopulations from healthy individuals and SLE patients were purified from the PBMCs by using magnetic beads. The relative mRNA expression of PRL receptor was determined in (A) T_reg_ and (B) T_eff_ cells by PCR-RT. Flow cytometry was used to determine the protein expression in (C) T_reg_ and (D) T_eff_ cells. The graph shows the median value.

Our results showed that T_reg_ cells from SLE patients express the PRL receptor even in absence of stimuli and that both mRNA (relative expression) and protein (FMI = mean fluorescence intensity) expression by T_reg_ cells from active and inactive SLE patients was higher than that in T_reg_ cells from healthy individuals (Table 1). This result showed a statistically significant difference (Figure 2 A and B), although no statistically significant difference were observed between the active and inactive patients. We found that the expression of PRL receptor mRNA and protein in T_eff_ cells from active and inactive SLE patients was higher than that in cells from healthy individuals, with a statistically significant difference (Figure 2C and D). There was no difference in the expression of PRL receptor between active and inactive patients. Moreover, the expression of PRL receptor was higher in T_reg_ cells compared to T_eff_ cells from patients with inactive SLE, similar to that observed in healthy individuals. However, in patients with active SLE, there was no difference in the expression of the receptor between T_reg_ and T_eff_ cells and the expression of the PRL receptor in T_eff_ cells from patients with SLE was higher than in healthy controls (Figure 2).

**TABLE 1:**
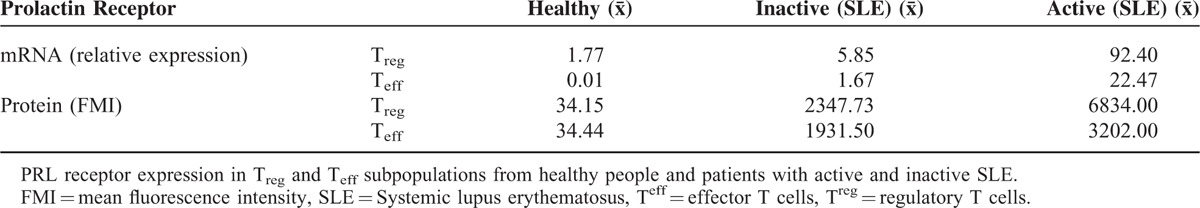
Expression of Prolactin Receptor

### PRL Function Regarding T_reg_ Cell-Meditated Regulation

**FIGURE 3:**
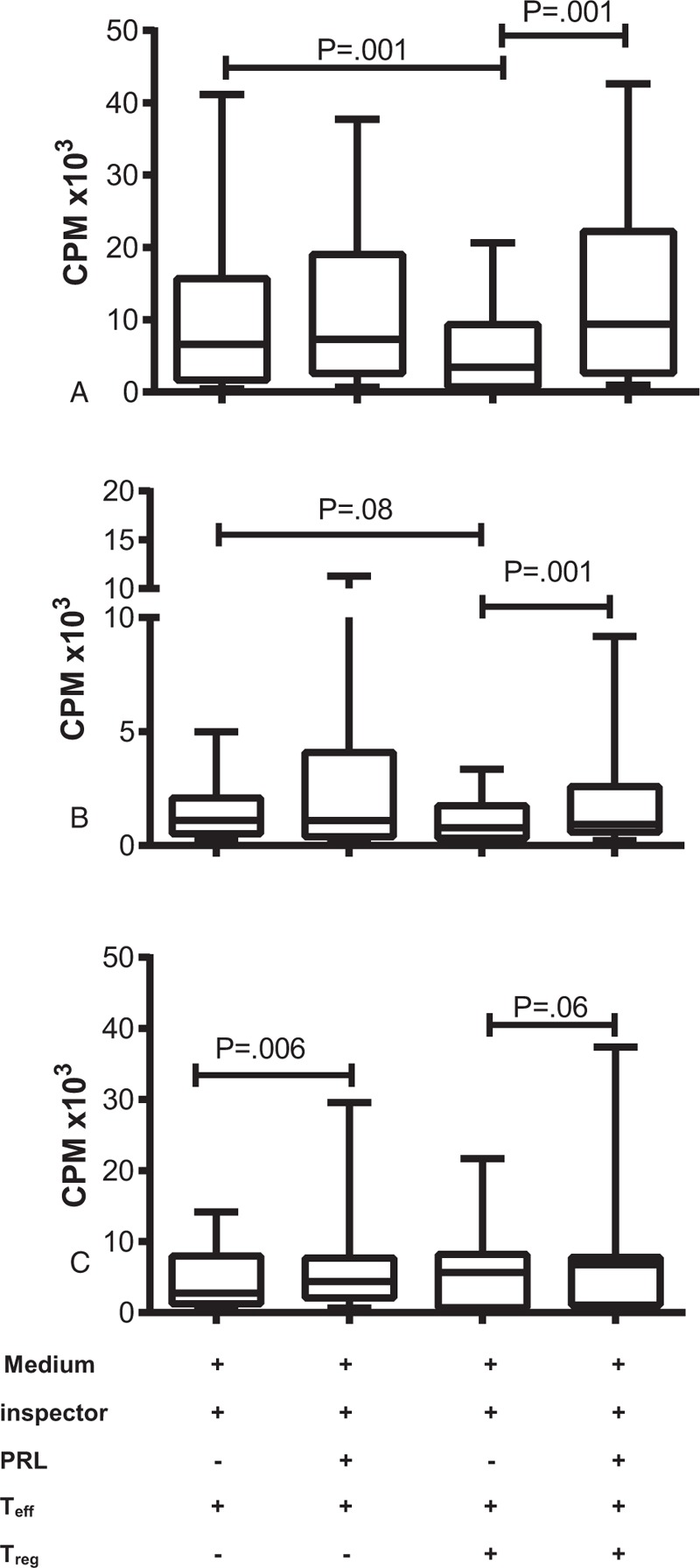
Effects of PRL on the functions of T_eff_ and T_reg_ cells, T_eff_ (CD4^+^CD25^−^CD127^+^) and T_reg_ (CD4^+^CD25^hi^CD127^−/low^) cells from healthy individuals and SLE patients were stimulated with “T_reg_ Suppression Inspector human” (anti-CD2/CD3/CD28 beads) in the presence and absence of PRL (50 ng/ml). Cell proliferation was measured by incorporating [^3^H]-thymidine in the cells from (A) healthy individuals, (B) inactive SLE patients, and (C) active SLE patients. The median value of 12 independent trials for each group is presented. The assays were performed in triplicate (statistical significance, *P* ≤ 0.05).

The suppressor capacity of T_reg_ cells stimulated with “T_reg_ Suppression Inspector human” (anti-CD2/CD3/CD28 beads) in the presence and absence of PRL was evaluated through in vitro cellular proliferation studies. The proliferation of T_eff_ cells from healthy individuals is shown in Figure 3A. We observed that the addition of PRL did not exert any effect on the proliferation of these cells when T_reg_ cells were added (coculture T_reg_:T_eff_), but the cells exerted suppressor activity over T_eff_ cells by decreasing their proliferation in a significant manner (*P* = 0.001). The addition of PRL to this coculture interfered with the activity of T_reg_ cells, reestablishing the proliferative capacity of T_eff_ cells to levels similar to that of T_eff_ cells in the absence of T_reg_ cells. PRL did not affect the proliferation of T_eff_ cells from patients with inactive SLE. The suppressor effect exerted by T_reg_ cells over T_eff_ cells was observed in most patients (Supplemental Content 2). However, when considering the entire group, we did not find any statistically significant difference (*P* = 0.08) in the suppressor effect of T_reg_ cells over T_eff_ cells. Similar to healthy subjects, PRL does not increase the proliferation of T_eff_ cells from inactive SLE patients. In T_reg:_T_eff_ coculture, the addition of PRL decreased the regulatory effect of T_reg_ cells, thus causing an increase in the proliferation of T_eff_ cells, with a statistically significant difference (*P* = 0.001; Figure 3B). In contrast, in cells from patients with active SLE, PRL activity increased the proliferation of T_eff_ cells in a statistically significant manner (*P* = 0.006). The T_reg_ cells from these patients did not have the capacity to exert their suppressor activity over the T_eff_ cells, although the addition of PRL to the T_reg_:T_eff_ coculture tended to increase the proliferation of T_eff_ cells, with no statistically significant difference (*P* = 0.06; Figure 3C). This result suggests that the function of T_reg_ cells is no longer adequate under this condition (Table 2).

**TABLE 2:**
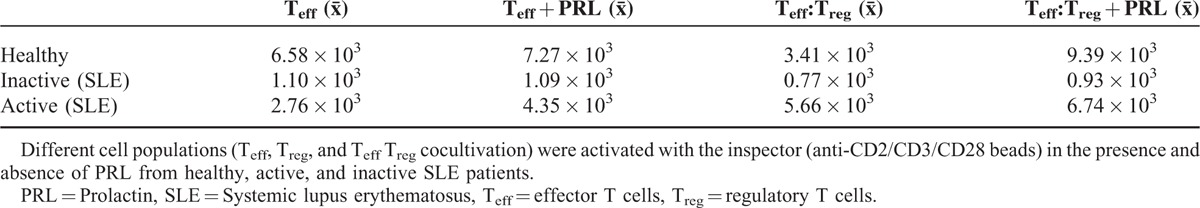
Cell Proliferation

### Cytokine Secretion by T_eff_ Cells Cultured in the Presence and Absence of PRL

**FIGURE 4:**
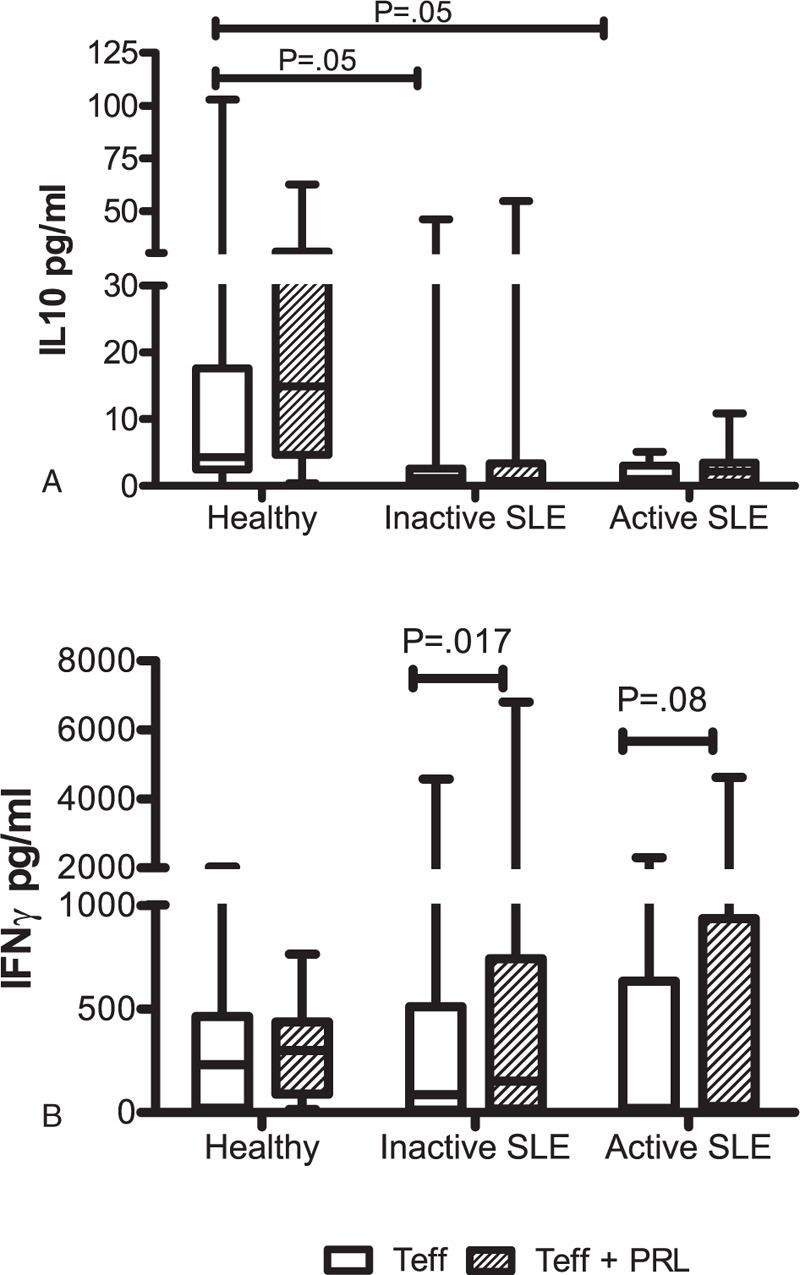
Cytokine secretion profile of T_eff_ in the presence and absence of PRL, T_eff_ cells from healthy persons and SLE patients were stimulated with “T_reg_ Suppression Inspector human” (anti-CD2/CD3/CD28 beads) in the presence and absence of PRL. The secretion of (A) IL10, and (B) IFNγ was determined by CBA. The median value is presented for each group (statistical significance, *P* ≤ 0.05).

Cytokine concentrations were determined in T_eff_ culture supernatants stimulated with “T_reg_ Suppression Inspector human” (anti-CD2/CD3/CD28 beads) in the presence and absence of PRL. As shown in Figure 4A, we observed that IL10 secretion from T_eff_ cells from patients with active or inactive SLE was decreased with respect to that from the cells from healthy individuals, with a statistically significant difference (*P* = 0.05), but there were no differences between inactive and active patients. The addition of PRL to the T_eff_ cell culture did not modify the secretion pattern of IL10 when using cells from any of the 3 studied groups. No difference in the secretion of IL17A, TNF, or IFNγ was observed for the T_eff_ cells from the 3 groups, and the addition of PRL did not affect IL17A and TNF secretion. However, PRL treatment increased IFNγ secretion from Te_ff_ from inactive patients, with a statistically significant difference (*P* = 0.01) (Figure 4B); meanwhile, in active patients, only an increase was observed, without any statistically significant difference (*P* = 0.08; Table 3).

**TABLE 3:**
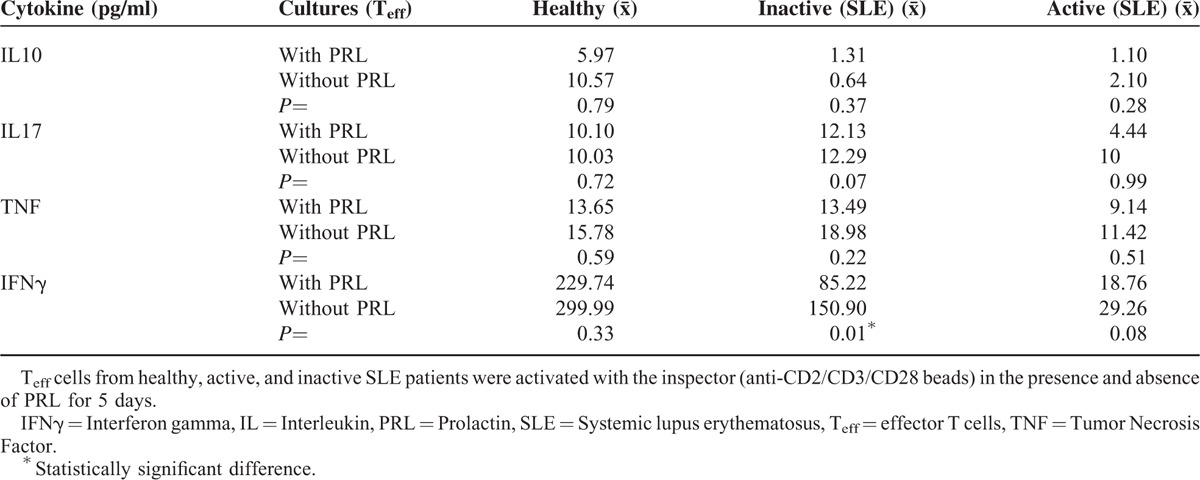
Cytokine Secretion by T_eff_ Cells

### Cytokine Secretion by T_reg_ and T_eff_ Cell Coculture in the Presence and Absence of PRL

**FIGURE 5:**
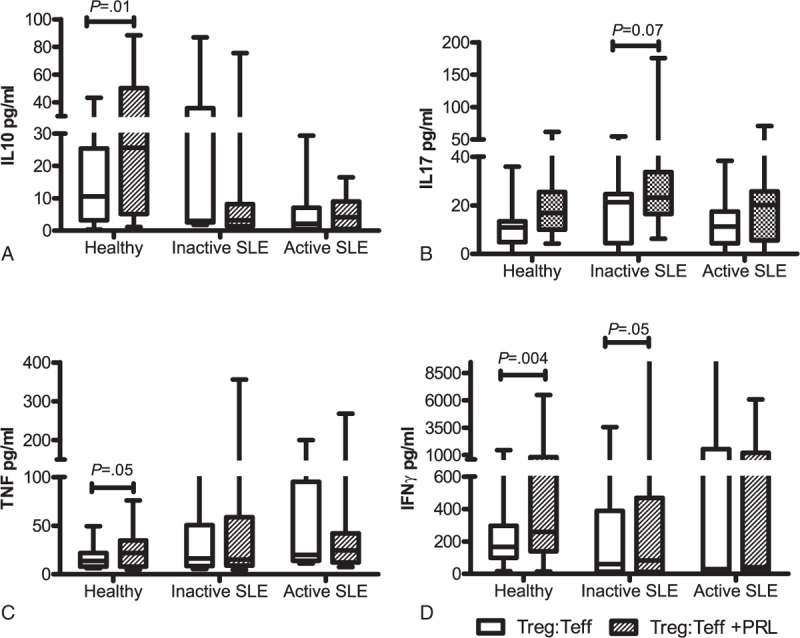
Cytokine secretion profile in T_reg_:T_eff_ coculture in the presence and absence of PRL, T_reg:_T_eff_ cocultures using cells from healthy persons and SLE patients were stimulated with “T_reg_ Suppression Inspector human” (anti-CD2/CD3/CD28 beads) in the presence and absence of PRL. The secretion of (A) IL10, (B) IL17, (C) TNF, and (D) IFNγ was determined by CBA. The median value is presented for each group (statistical significance, *P* ≤ 0.05).

Cytokine secretion was determined in the presence and absence of PRL by using T_reg:_T_eff_ cocultures from the 3 groups being studied. The addition of PRL to the T_reg:_T_eff_ coculture from healthy individuals significantly increased the secretion of IL10, TNF, and IFNγ, whereas IL17A secretion was unaffected. Meanwhile, PRL significantly increased IFNγ secretion in T_reg_:T_eff_ cocultures using cells from patients with inactive SLE (*P* = 0.05) and IL17A secretion increased in most patients. However, we did not find any statistically significant difference (*P* = .07) in case of the entire group; there was no difference in TNF and IL10 secretion. Cytokine secretion was not affected by the addition of PRL to the cocultures using cells from patients with active SLE (Figure 5, Table 4).

**TABLE 4:**
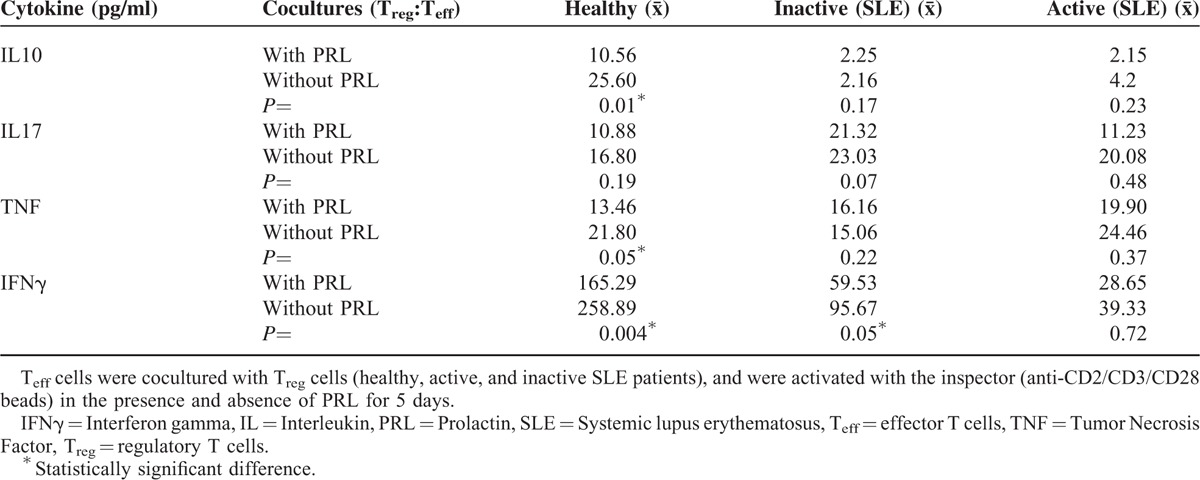
Cytokine Secretion by T_eff_:T_reg_ Cocultures

## DISCUSSION

Sex hormones such as PRL play an important role in the modulation of immune response, which depends on the type of cell expressing the PRL receptor.^7,34^ Moreover, PRL has an immune-stimulating effect and promotes autoimmunity,^5^ interfering with the tolerance of B cells^35^ and increasing the production of antibodies.^5,36^ We previously reported that the PRL receptor is constitutively expressed in the T_reg_ cells of healthy individuals (females), whereas the expression increases in T_eff_ cells in response to a stimulus.^13^ The results of this study showed that compared to healthy individuals, the expression of PRL receptor was higher in the T_reg_ and T_eff_ cells from patients with SLE (females), with the receptor being expressed even in the absence of a stimulus. This expression tended to increase in cells from active patients compared to that from inactive patients, suggesting higher activity in the disease, along with higher expression of the receptor. Which occurs in B cells from mice that developed lupus (MRL, MRL/lpr), whereby the expression of the receptor increased with the manifestation of the disease.^37,38^ In addition, the expression patterns of T_eff_ and T_reg_ cells differed between active and inactive patients. In inactive patients, the expression of the receptor was higher in T_reg_ cells compared to T_eff_ cells, a behavior similar to that observed in healthy individuals. However, there was no difference in the expression of the receptor between T_eff_ and T_reg_ cells from active patients, most likely because the T_eff_ cells were already active, increasing the expression of PRL receptor. This would be similar to the phenomenon in T_eff_ cells from healthy individuals: when activated, the cells increase the expression of the receptor to a level higher than that in T_reg_ cells.^13^

T_reg_ cells are a component of one of the peripheral tolerance mechanisms, which fail in autoimmune diseases such as SLE; therefore, these cells are important in the pathogenesis of the disease.^2^ However, available data on the number and function of T_reg_ cells in SLE are contradictory, and the definitive role of T_reg_ cells in SLE remains unclear.^29^ Therefore, we decided to explore, the percentage of T_reg_ cells in patients with active and inactive SLE, and the role played by PRL in the regulatory function of these cells ex vivo. A statistically significant decrease was found in the percentage of T_reg_ (CD4^+^CD25^hi^CD127^−/low^ FOXP3^+^) cells from patients with SLE, both active and inactive, compared to that in healthy individuals, supporting the findings of previous studies.^22,24,39,40^ Additionally, the suppressor function exerted by T_reg_ cells over T_eff_ cells depends on the stage of the disease. In patients with inactive SLE, we observed 2 behaviors; first, T_reg_ cells did not present any defects in their suppressor activity (majority of the patients), and second, T_reg_ cells did not present a suppressor function in another group of patients (minority of the patients). Although the patients are clinically inactive, their immune system is probably active, and therefore, T_reg_ cells no longer exert their suppressor effect, as observed in active patients where we did not observe T_reg_ suppressor function, as has been reported. The decrease in the number and function of T_reg_ cells in SLE patients favors the activation of autoreactive clones, and thus, disease manifestation.^26,40,41^

Because T_reg_ cells from SLE patients express high levels of PRL receptor, we studied whether an interaction with its PRL receptor could affect the suppressor effect of T_reg_ cells, especially those from inactive patients, possessing suppressor function. In these patients, PRL blocked the suppressor effect of T_reg_ cells on T_eff_ cells, a behavior similar to healthy individuals.^13^ The loss of suppressor effect cannot be attributed to the notion that PRL increases the proliferation of T_eff_ cells, because the addition of PRL to the T_eff_ cell culture did not increase the proliferation of these cells. It might be due to the presence of proinflammatory cytokines (IFNα, IFNγ, and TNF),^42–45^ as their presence in the culture reduces the suppressor effect of T_reg_ cells. It is also known that PRL promotes the secretion of cytokines such as IFNγ, IL2, IL12, and TNF.^12,46,47^ Our results showed an increase in IFNγ levels in the cocultures incubated with PRL (T_reg_:T_eff_ of inactive patients), and although an increase in IL17 levels was observed in these cultures, it was not statistically significant. The increase in IFNγ levels by the addition of PRL was also observed in T_eff_ cell cultures (expressing PRL receptor), which makes us hypothesize that interaction of PRL with its receptor on T_eff_ cells increases IFNγ secretion, and that the presence of this cytokine in the culture decreases the suppressor function of T_reg_ cells in patients with inactive SLE, because this cytokine is known to inhibit the generation and/or function of T_reg_ cells.^44,48,49^ It is also possible that IFNγ is secreted by T_reg_ cells, as reported in patients with type I diabetes and rheumatoid arthritis, diseases in which T_reg_ cells that secrete proinflammatory cytokines as IFNγ and IL17.^50–52^ Unfortunately because of the low number of T_reg_ cells purified from patients, we could not verify whether PRL favors IFNγ secretion in these cells. It will be interesting to show whether PRL favors the presence of T_reg_ IFNγ-secreting cells, especially because this has been reported for other autoimmune diseases.^50–52^

Our results show that both T_reg_ and T_eff_ cells in women with inactive SLE constitutively express the PRL receptor, and therefore, an increase in serum PRL levels will favor the interaction of PRL and its receptor and, in turn, the malfunctioning of the Treg cells, probably because of presence of IFNγ. This malfunction, added to the decrease in the cell number, will contribute to the expansion of autoreactive T-lymphocytes, favoring disease activation. In patients with active SLE, different from those with inactive SLE, PRL increased the cellular proliferation of T_eff_ cells. Thus, PRL in active patients could help in maintaining the disease active by favoring the proliferation of T_eff_ cells among those that are autoreactive.

It is worth mentioning that in our study, we did not use antigen-presenting cells (APCs); only T_reg_ cells were coincubated with T_eff_ cells to observe the suppressor effect of T_reg_ cells. Other models using APCs as a suppressor of the function of T_reg_ cells have been reported. In this sense, it has been proposed that the APCs can block T_reg_ cell activity via overproduction of pro-inflammatory cytokines such as IFNα.^42^ It would be interesting to determine whether APCs express PRL receptor, and whether PRL favors the secretion of IFNα and other inflammatory cytokines, thereby aiding the malfunction of T_reg_ cells in SLE patients.

## CONCLUSIONS

Our results showed that T_reg_ cells from patients with SLE differed from those from healthy individuals with regard to number and function. In inactive patients, PRL acts on T_eff_ cells, which constitutively express the receptor, increasing IFNγ secretion and encouraging an inflammatory microenvironment and T_reg_ cell malfunction. The decrease in the number of T reg cells and their malfunction can contribute to the expansion of autoreactive T-lymphocytes to favor disease activation. Additionally, in active patients, PRL increases the proliferation of inspector-stimulated T_eff_ cells, which can further aid the T_eff_ cells to be more resistant to regulation by T_reg_ cells. It will be interesting to study whether PRL decreases the function of different subpopulations of T_reg_ cells and whether this decrease occurs because PRL favors the plasticity of T_reg_ cells toward a Th1 profile.

## Supplementary Material

Supplemental Digital Content
