# Supplementary material for: Function of Treg Cells Decreased in Patients With Systemic Lupus Erythematosus Due To the Effect of Prolactin
Source: Medicine (Baltimore). 2016 Feb 8;95(5):e2384. doi: 10.1097/MD.0000000000002384 (PMC4748869; doi:10.1097/MD.0000000000002384)

Supplemental Content 1. Purification of T_reg_ and T_eff_ cells.

T_reg_ and T_eff_ cells were isolated from PBMCs by using a CD4^+^ CD25^+^CD127^dim/^, Regulatory T cell Isolation Kit II. The purity of the cells ranged between 93% and 97%.


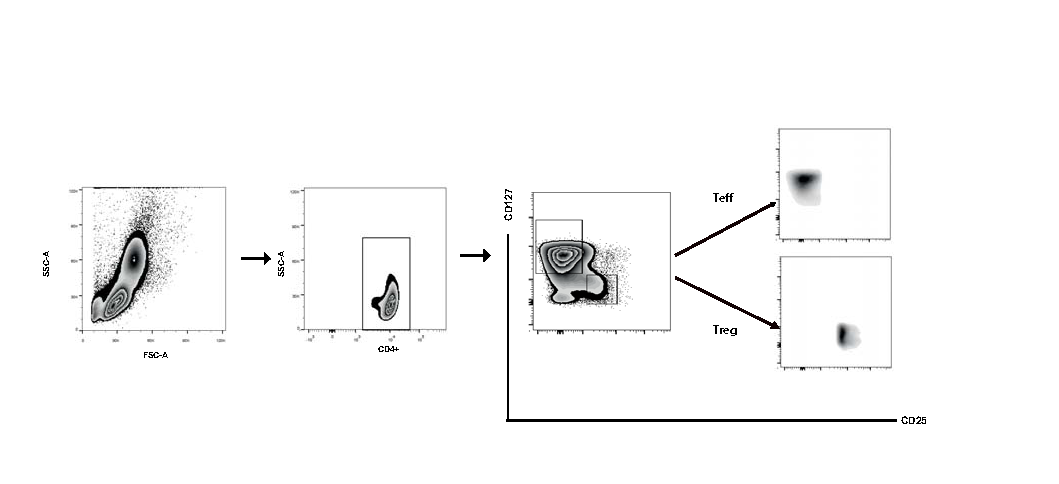


Supplemental Content 2. Suppressor effect exerted by T_reg_ cells over T_eff_ cells, as observed in most inactive SLE patients.

T_reg_ (CD4^+^CD25^hi^CD127^low/-^) and T_eff_ (CD4^+^CD25^-^CD127^+^) cells from inactive SLE patients were stimulated with ``T_reg_ Suppression Inspector human'' (anti-CD2/CD3/CD28 beads) in the presence and absence of PRL (50░ng/ml). Cell proliferation was measured by the incorporation of [^3^H]-thymidine. The assays were performed in triplicate.


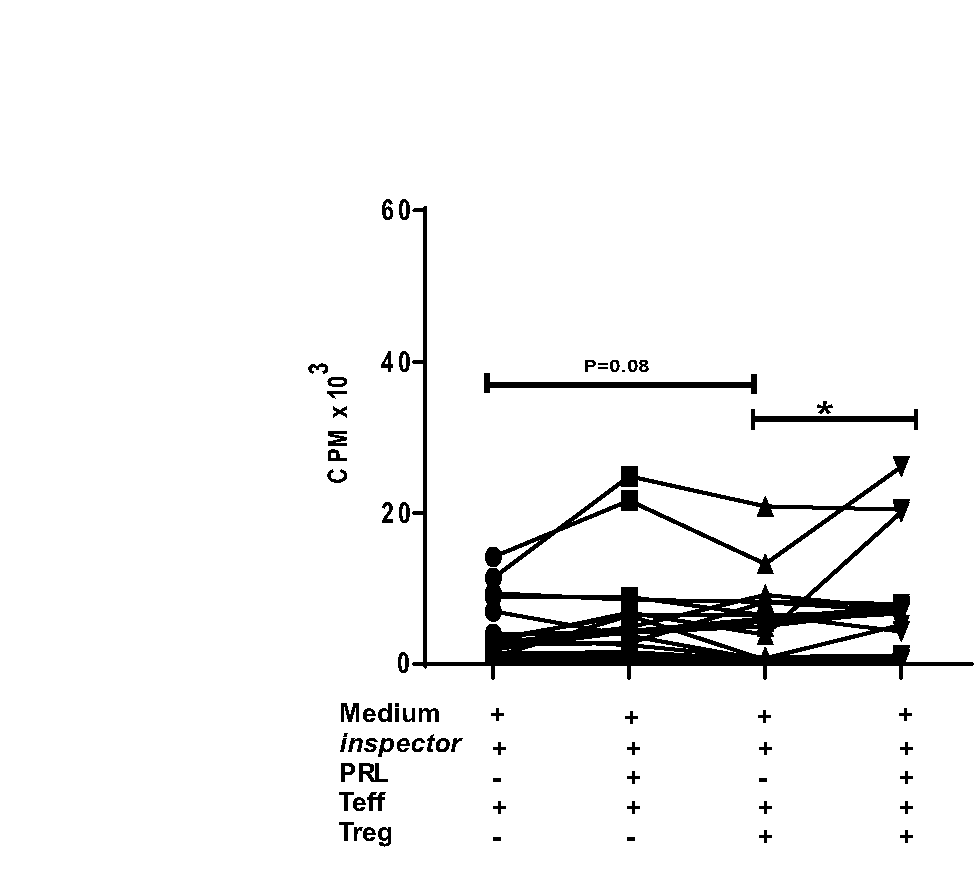

Supplement: Supplemental Digital Content [file medi-95-e2384-s001.doc]
